# Prebiotic Sugar Formation Under Nonaqueous Conditions and Mechanochemical Acceleration

**DOI:** 10.3390/life9020052

**Published:** 2019-06-20

**Authors:** Saskia Lamour, Sebastian Pallmann, Maren Haas, Oliver Trapp

**Affiliations:** 1 Department Chemie, Ludwig-Maximilians-Universität München, Butenandtstr. 5-13, 81377 München, Germany; saskia.lamour@cup.lmu.de (S.L.); sebpall@aol.com (S.P.); maren.haas@cup.lmu.de (M.H.); 2 Max-Planck-Institut für Astronomie, Königstuhl 17, 69117 Heidelberg, Germany

**Keywords:** aldol reaction, mechanochemistry, minerals, monosaccharides, prebiotic chemistry

## Abstract

Monosaccharides represent one of the major building blocks of life. One of the plausible prebiotic synthesis routes is the formose network, which generates sugars from C1 and C2 carbon sources in basic aqueous solution. We report on the feasibility of the formation of monosaccharides under physical forces simulated in a ball mill starting from formaldehyde, glycolaldehyde, DL-glyceraldehyde as prebiotically available substrates using catalytically active, basic minerals. We investigated the influence of the mechanic energy input on our model system using calcium hydroxide in an oscillatory ball mill. We show that the synthesis of monosaccharides is kinetically accelerated under mechanochemical conditions. The resulting sugar mixture contains monosaccharides with straight and branched carbon chains as well as decomposition products. In comparison to the sugar formation in water, the monosaccharides formed under mechanochemical conditions are more stable and selectively synthesized. Our results imply the possibility of a prebiotic monosaccharide origin in geochemical environments scant or devoid of water promoted by mechanochemical forces such as meteorite impacts or lithospheric activity.

## 1. Introduction

**Scheme 1:**
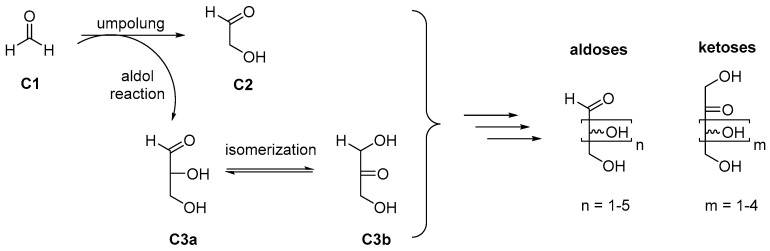
Monosaccharide synthesis in the formose reaction network; **C1**: formaldehyde, **C2**: glycolaldehyde, **C3a**: glyceraldehyde, **C3b**: dihydroxyacetone.

The formose reaction is the classical route to carbohydrates based on the oligomerization of formaldehyde (C1) through a cascade of cross-, retro- and aldol reactions in aqueous solution in the presence of a basic catalyst, typically calcium hydroxide (Scheme 1) [1,2,3,4]. The resulting product mixture contains sugars of different length, constitution, and configuration [5,6,7,8]. The missing selectivity for specific monosaccharides is one of the major problems of the formose reaction in the context of the origin of life. Furthermore, isomerization and the instability of sugars under basic conditions are obstructive to the sugar formation in aqueous solutions [9,10]. The subsequent degradation reactions produce, for example, lactic acid, saccharic acid and α-dicarbonylic acids [11] and result in a dark tar-like substance. A water-independent reaction pathway to monosaccharides offers a consequential approach.

Contemplated scenarios for the origin(s) of life are as ample as the variety of building blocks, and molecules life is made of [12]. Examples of those are the warm little pond [13], hydrothermal vents [14,15], volcanic environments [16,17], drying lagoons [18,19,20,21], the primordial soup [22,23], eutectic solutions [24,25] or comet ponds [26,27,28]. Whereas each of them addresses distinct open questions in the context of the emergence of life, certain issues remain unresolved. A particular one lies with the contradictory assumption that life was formed in water when several chemical and biologically relevant transformations are condensation reactions that are disfavored in aqueous environments [29,30]. In this respect, only a scarce number of mechanochemical approaches [31,32] have been identified so far that plausibly demonstrate the formation of biologically relevant molecules when only grinding or milling of dry substrates is used to trigger reactivity. Such examples include the synthesis of α-amino acid derivatives [33,34] and modification of nucleosides and nucleotides [35]. Sources for mechanochemical energy considered are lithospheric activities such as weathering, erosion, diagenetic processes and meteoritic impacts on Earth. They can also be accounted for in asteroids tectonics [36]. Based on our interest in the formose reaction network [37] and on the feasibility of aldol reactions in mechanochemical setups [38,39], we investigated the potential formation of carbohydrates under nonaqueous and mechanochemical reaction conditions. We used an oscillatory and a planetary ball mill, which have a high mechanic input leading to shorter and therefore more practicable reaction times.

## 2. Materials and Methods

All chemicals were used as received. *O*-ethylhydroxylamine hydrochloride (99%) *N*,*O*-bis(trimethylsilyl)trifluoroacetamide (BSTFA) (99%), pyridine (99%), phenyl-β-D-glucopyranoside (97%), paraformaldehyde (95%), glycolaldehyde (as dimer) (mixture of stereoisomers) and DL-glyceraldehyde (as dimer) (95%) were purchased from Sigma-Aldrich or TCI Germany GmbH. Calcium hydroxide (p.a.) was supplied by the chemical store of the Faculty for Chemistry and Pharmacy of the Ludwig-Maximilian University Munich, Germany. Portlandite and brucite were purchased from Seltene Mineralien, Gunnar Färber, Samswegen. Sodium montmorillonite clay was received from ABCR. Molecular sieves (4 Å, Type 514, pearl form) were acquired from Carl Roth GmbH & Co., KG. Water was deionized (DI) by a VWR Puranity PU 15 (VWR, Leuven, Belgium).

Adsorbed formaldehyde was prepared as follows: Anhydrous paraformaldehyde was heated under nitrogen flow to 150 °C and the gaseous monomer led through a column filled with dry molecular sieves (0.4 nm). The resulting molecular sieves contained approximately 12.5 wt% adsorbed formaldehyde, determined by weight increase.

For sugar formation under nonaqueous conditions, C2 (1.25 g, 20.8 mmol, 1.00 eq) and Ca(OH)_2_ (310 mg, 4.18 mmol, 0.20 eq) were mixed using a vortex mixer in a 10 mL glass vial. In 2 mL glass vials, reaction mixtures of 156 mg were placed in an air-conditioned room at 23 °C.

The mechanochemically promoted synthesis of carbohydrates was conducted following these procedures: A 5 mL stainless steel ball mill jar was either charged with a) 125 mg C2 (2.08 mmol, 1.00 eq) and 31 mg Ca(OH)_2_ (0.42 mmol, 0.20 eq) or b) 52 mg C2 (0.86 mmol, 0.50 eq), 78 mg C3a (0.86 mmol, 0.50 eq) and 26 mg Ca(OH)_2_ (0.35 mmol, 0.20 eq). Reactions with mineral catalysts were also performed using 20 mol% catalyst and the same total mass, approximately 155 mg. The reaction mixtures were immediately ground using a single 7 mm stainless steel ball in the oscillatory ball mill CryoMill (Retsch GmbH, Haan, Germany) at a frequency of 30 Hz. Reactions with formaldehyde were conducted in 20 mL stainless steel grinding bowls with ten 10 mm balls. 1.0 g formaldehyde-loaded molecular sieves (125 mg formaldehyde, 4.16 mol, 0.50 eq.), 250 mg C2 (4.16 mmol, 0.50 eq.) and 123 mg Ca(OH)_2_ (1.67 mmol, 0.20 eq.) were added, the jar flushed with nitrogen gas and immediately grinded in the Pulverisette 7 (Fritsch GmbH, Idar-Oberstein, Germany) at 400 rpm for 90 min.

For the reaction in aqueous solution, C2 (140 mM) was dissolved in degassed water at 40 °C, and 20 mol% Ca(OH)_2_ was added.

If not analyzed immediately, samples were stored at −196 °C in liquid nitrogen to prevent further reaction. Aqueous solutions were lyophilized prior to derivatization.

GC-MS detection of carbohydrates followed a published protocol [40]. In short: About 2 to 5 mg of the sample was dissolved in 200 µL pyridine, mixed with 200 µL of a 40 mg/mL *O*-ethylhydroxylamine hydrochloride solution with 50 mM phenyl-*β*-D-glucopyranoside as internal standard and heated for 30 min at 70 °C on a rocking shaker. To this mixture, 120 µL BSTFA was added and the resulting solution was heated again for 30 min at 70 °C. The derivatized sugars were separated by GC-MS on a TraceGC Ultra system coupled to a PolarisQ MS (quadrupole-ion trap mass spectrometer [MS]) operated by Xcalibur software. Injections were performed using a split/splitless injector in split mode at 250 °C. Flame ionization detection was co-recorded with MS data and operated under carbon-correction at 250 °C. A SE-52 column (14 m length, ID 250 nm, 250 nm film thickness) with 80 kPa helium was used with the following temperature program: Beginning at 50 °C for 2 min and increasing temperature by 10 K/min to 140 °C and then 5 K/min to 240 °C and keeping that temperature for 2 min. Due to *E/Z*-isomerism of oximes, carbohydrate analytes can show two signals.

FID peak areas of respective carbohydrates (C2–C7) were corrected by their effective carbon number to account for different response factors. This number has been calculated for each derivatized sugar and the internal standard from literature values for individual functional groups [41]. For relative compositions, each sugar value was put in proportion with all relevant sugar groups. For kinetic studies, the absolute concentration of the derivatized sample was determined with the help of calibration lines and area ratios in relation to the internal standard. This composition was transferred to the molecular distribution of the mechanochemical approach. Errors of relative frequencies are standard deviations of duplicate experiments and duplicate or triplicate derivatization procedures.

## 3. Results and Discussion

### 3.1. Mineral-Catalyzed Mechanochemical Monosaccharide Synthesis

**Figure 1:**
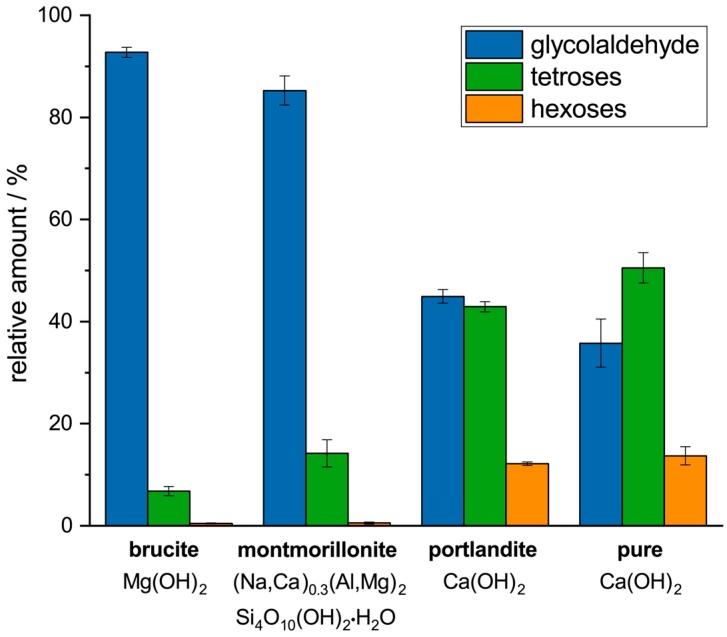
Comparison of different mineral catalysts with pure calcium hydroxide used in mechanochemical sugar formation starting from glycolaldehyde. Oscillatory ball mill, 30 Hz, 90 min, 20 mol% catalyst.

In classic organic chemistry, the formose reaction starts with formaldehyde (plus glycolaldehyde) and a base, most often calcium hydroxide, in aqueous solution. Since formaldehyde (C1) itself is gaseous and solid only below −92.15 °C, it is not easily deployable for solid phase reactions. Alternatively, the polymerized forms paraformaldehyde and trioxane could be used. However, neither of them depolymerized under the chosen conditions and were, therefore, unreactive. As a result, we employed glycolaldehyde (C2) and DL-glyceraldehyde (C3a) as smallest carbohydrate building blocks. Both have been shown to be prebiotically relevant and are connected to terrestrial and extra-terrestrial origins [42,43,44,45,46,47]. A plethora of minerals was already abundant on the early Earth during the Hadean Eon, the era when life and its building blocks is believed to have formed [48]. These represent possible catalysts for prebiotic reactions and have been shown to be active in aqueous formose settings conducted under the aspect of the origin(s) of life [49,50]. As basic catalysts are most active and the common catalyst is calcium hydroxide, three hydroxyl minerals were chosen: portlandite, brucite, and montmorillonite. Knowing about mechanochemical approaches in the context of aldol reactions [31,38,39,51,52,53], we tested ball milling for its ability to promote and direct the sugar formation. Upon ball milling glycolaldehyde with 20 mol% mineral additive (30 Hz, 90 min) and analysis of the product mixture after derivatization by GC-MS, we found the formation of tetroses and hexoses in all three cases (Figure 1). The reactions with brucite and montmorillonite only showed traces of hexoses, but (6.79 ± 0.93)% and (14.19 ± 2.66)% tetroses, respectively. The highest conversion was observed with portlandite (73%) yielding (42.91 ± 0.99)% tetroses and (12.16 ± 0.35)% hexoses. In comparison with pure calcium hydroxide, the product contribution is similar, but the conversion is slightly lower. After establishing the ability to form higher monosaccharides with minerals, further investigations were conducted with pure calcium hydroxide as a model catalyst.

### 3.2. Model System: Nonaqueous Reaction

In a first instance, we investigated the reactivity of glycolaldehyde in the presence of 20 mol% calcium hydroxide at room temperature over the course of a month without any mechanical impact. The mixed solids were let to rest in a glass vial for a defined time interval before immediate derivatization and analysis. Already after 24 hours, we observed the formation of tetroses, namely erythrose, threose, and erythrulose, making up (1.12 ± 0.05)% of the reaction mixture. At further reaction progress, the consumption of glycolaldehyde increases while tetroses and hexoses form.

After 28 days, the initially colorless, powdery mixture turns into a yellow, viscous liquid with hexoses making up (66.36 ± 0.37)%. The change of appearance is typical for formose-type reaction of carbohydrates and is known as the yellowing point. This is when carbohydrates of higher order and complex branching occur. Further reaction results in an only partly solvable tar. Right before this point, we observe heptoses—the highest order carbohydrates resolvable by the here employed GC-MS method. The generation of heptoses is an indication of reversible reaction pathways since formal additions of C2 and multiples thereof can only result in even number carbon skeletons. Therefore, a growing proportion of retro-aldol reactions of long-chained carbohydrates can be considered.

We also investigated the temperature dependence for the initial sugar formation and found that elevated temperatures of (a) 50 °C and (b) 60 °C promote a significant monosaccharide formation within half an hour. Relative frequency for tetroses and hexoses were (a) (7.14 ± 0.23)% and (0.88 ± 0.03)% and (b) (38.38 ± 0.90)% and (5.10 ± 0.25)%, respectively. On the other hand, low temperatures (<0 °C) prevented reactivity and were, therefore, used for storage. Higher temperatures than those mentioned were not investigated due to the low melting point of glycolaldehyde. Further data on the temperature dependence are part of the Supplementary Materials.

### 3.3. Model System: Kinetic Investigations of Mechanochemical Reaction

**Figure 2:**
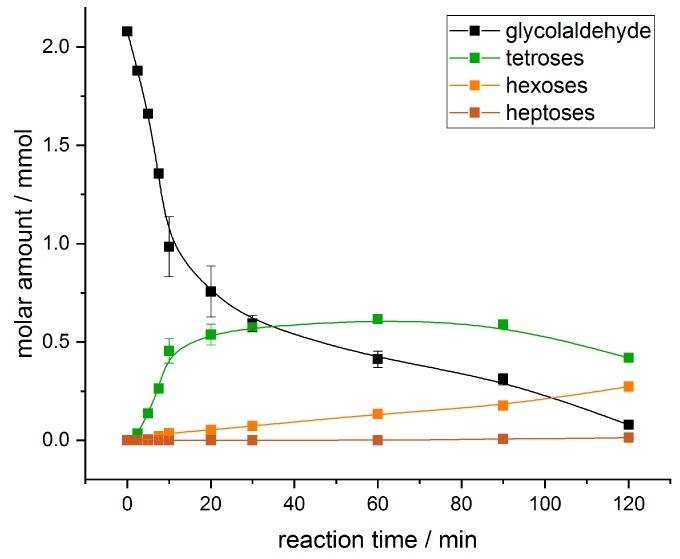
Reaction progress of glycolaldehyde and 20 mol% calcium hydroxide in an oscillatory ball mill at 30 Hz up to 120 min.

**Figure 3:**
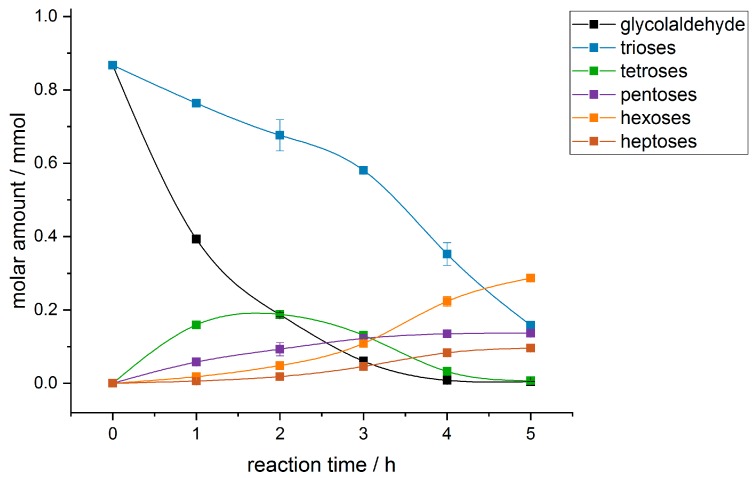
Reaction progress of glycolaldehyde and glyceraldehyde (1:1) plus 20 mol% calcium hydroxide in an oscillatory ball mill at 30 Hz up to 5 h.

After substantiating the feasibility of carbohydrate synthesis under dry conditions without any energy source, we proceeded to examine the influence of a mechanic energy source instead of temperature. In our experiments, we used an oscillatory ball mill charged with I) **C2** or II) **C2** and C3a together with Ca(OH)_2_ (Figure 2 and Figure 3). We made sure that for comparability the employed mixtures were of the same total mass. Thus, momentum transfer is equal. Grinding was performed at 30 Hz and did not cause a significant increase in temperature. On average, one hour of grinding resulted in a temperature rise of 1 K. Catalyst loading was 20 mol%. We also tested the feasibility of lower catalyst loading. It was found that it slowed down the reaction rate. For further details, see Supplementary Materials.

In both cases I) and II), we observed that carbohydrate synthesis is achievable and that the reaction rate is accelerated. Results are depicted in Figure 2 and Figure 3. In order to verify the hypothesis that grinding promotes the sugar formation through energy transfer rather than by efficient blending of the reactants, we milled the glycolaldehyde samples at 2.5, 5, 7.5 and 10 min and derivatized them all simultaneously after 10 minutes in total, so that, for instance, the sample of 2.5 min was kept for 7.5 min etc. If efficient intermixing was the sole contributor for an enhanced reactivity, all samples should show the same product distribution as within a minute of milling the substrates are already mixed effectively. However, we observed an exponential consumption of glycolaldehyde instead and, thus, conclude a kinetic acceleration by the mechanochemical energy. In fact, for longer reaction times, no significant dependence between reaction progress and milling was found. We explain this observation with a changing aggregate state of the mixture. With proceeding milling time, the reaction mixtures turn, firstly, from colorless powders into yellow viscous pastes before, secondly, they eventually become dark yellow solids that are not completely dissolved in our derivatization solutions. The kinetic data were only recorded up to this point (I) 2 h, II) 5 h). For longer reaction times the derivatized samples still contained the described monosaccharides, undissolved tar was not further investigated. During the first transition of texture, milling becomes ineffective as the sticky paste decelerates the ball within the milling jar and thus reduces energy transfer. At this time, the reaction progress is mainly diffusion controlled and is not accompanied by effective blending. As the reactants are already thoroughly mixed and in close contact in the viscous solution, the reaction rate is still sufficiently high. With further reaction progress, the mixture solidifies and becomes responsive to grinding again.

For the reaction I) starting from glycolaldehyde the course of the reaction develops as follows: Glycolaldehyde was consumed rapidly leading to the bisection of its amount after 10 min. Meanwhile tetroses were formed with a slightly slower rate. In the next ten minutes, the rate of glycolaldehyde consumption slowed down to 1/16 of the starting rate, the tetrose amount stabilized and hexoses increased steadily. After 90 min, due to less abundance of glycolaldehyde as feedstock for tetroses, their amount started to decrease in favor of hexoses. Conversion of glycolaldehyde was near to complete after 120 min. Tetroses and hexoses were present in similar amounts by that time and heptoses occurred in minor amounts.

The product distributions for milled and not milled reactions are similar but not equal. For the final measured data points (28 d in a vial vs. 120 min milling), the mechanochemical reaction sample consists of about 3.7 times more tetroses while having the same glycolaldehyde, half the hexoses and one-fifth of the heptoses abundance in relative ratios. This approach, thus, exhibits a different selectivity in favor of shorter carbohydrates. Interestingly, also heptoses are formed probably due to similar reasons as already explained for the non-mechanochemical solid phase reaction of glycolaldehyde. 

For increasing the complexity of the reaction network, we further investigated the mechanochemically accelerated reaction of glycolaldehyde with calcium hydroxide in the presence of glyceraldehyde. This way, also pentoses and heptoses are accessible by direct aldol condensation reactions. As it can be inferred from Figure 3, glycolaldehyde consumption is considerably faster (3.6 times) than the one of the trioses. Glyceraldehyde is only being significantly used up after glycolaldehyde is almost 10 times less available. At this time point, the reaction rate of hexoses increased more than trifold, whereas tetroses were abundant in the experiments discussed previously, in the presence of trioses they are effectively consumed after their maximum abundance at 2 h for the sake of heptoses. Moreover, dihydroxyacetone is formed during the cause of the reaction, which likely is both the result of enediolization of glyceraldehyde and retro-aldol reactions (Supplementary Materials). In contrast to glyceraldehyde being consumed, dihydroxyacetone is enriched over the cause of the reaction.

In the final product mixture, glycolaldehyde and tetroses were nearly fully converted into higher monosaccharides. Up to the extent of our kinetic studies, triose content decreased down to a fifth of the starting amount. Similar amounts were found for pentoses and heptoses. The major components of this final product distribution were hexoses as a statistic result of having two possible reaction paths generating them: C2 + C2 + C2 and C3 + C3.

**Figure 4:**
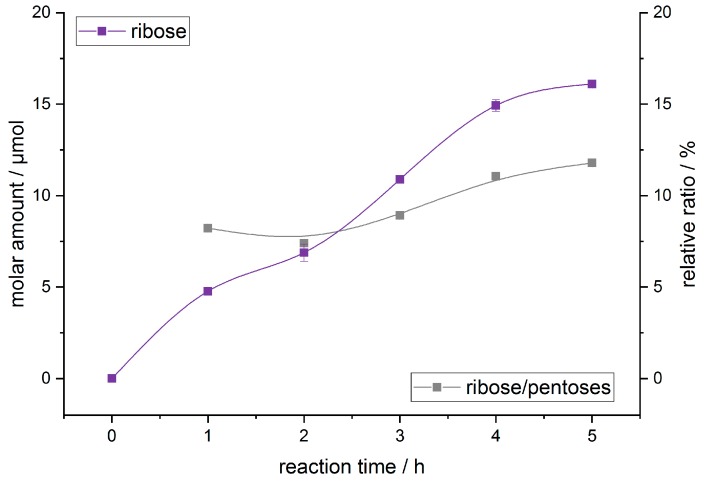
Synthesis of ribose and the relative ratio of ribose relating to all pentoses during the reaction of glycolaldehyde and glyceraldehyde (1:1) with 20 mol% calcium hydroxide in an oscillatory ball mill at 30 Hz up to 5 h.

The reaction mixture consists of unbranched aldoses and ketoses and a minor amount of branched monosaccharides, but only few decomposition products. In comparison with commercially purchased samples, several monosaccharides were identified from the mixture, namely xylose, lyxose, arabinose, xylulose, ribulose, ribose, apiose, tagatose, psicose as well as sorbose and/or fructose (latter two not separated by GC-MS). As a fundamental monosaccharide for life, ribose was synthesized during the mechanochemical reaction. Ribose was formed in 2% yield and in comparison, the relative ratio of ribose to all pentoses increases over the course of reaction up to 12% after 5 h (Figure 4).

### 3.4. Comparison with Aqueous Reaction (C2+C2)

**Figure 5:**
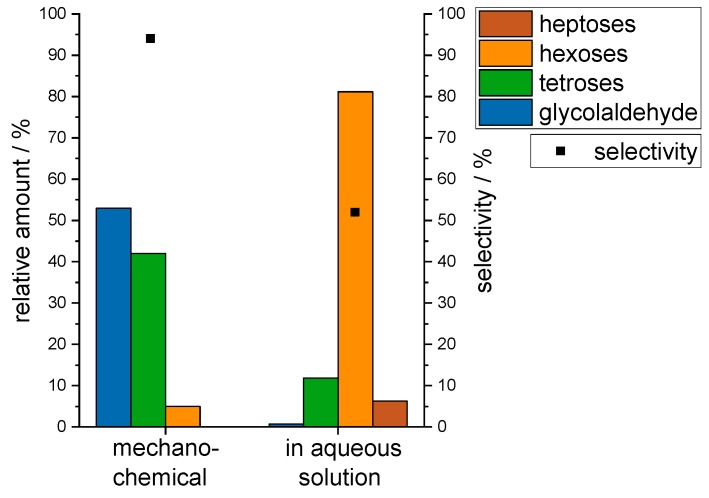
Composition of monosaccharides and selectivity of the aldol reaction using glycolaldehyde, catalyzed by 20 mol% calcium hydroxide, after 30 min under two different reaction conditions. Mechanochemical: oscillatory ball mill, 30 Hz. In aqueous solution: 140 mM glycolaldehyde, 40 °C.

The mechanochemical reaction was compared to its counterpart reaction in water starting with glycolaldehyde (140 mM) and calcium hydroxide at 40 °C (Figure 5). After 30 min glycolaldehyde was almost completely consumed for the higher monosaccharide synthesis. Hexoses represent the major proportion of the monosaccharides. Although the conversion was accelerated, the selectivity towards aldoses und ketoses was decreased and reached only 50%–60% with decomposition products like lactic acid taking up a substantial part of the product mixture. In the mechanochemical set-up, the reaction was much slower. Due to the deceleration, the decomposition is also delayed and selectivities around 95% are generated. In the context of the origin of life, the nonaqueous reaction supplies a variety of sugar building blocks and feedstock molecules for subsequent reactions towards biologically relevant molecules. As a result of the selectivity and decelerated formation, higher monosaccharides remain available to a higher degree for further reaction steps.

### 3.5. Built-In of Formaldehyde

To investigate the reactivity of the C1 building block formaldehyde, the gaseous reactant needs to be made accessible for ball-milling. Depolymerization of paraformaldehyde or trioxane in the ball mill was not feasible in this reaction setup. Instead, availability of monomeric formaldehyde was achieved by thermal depolymerization of paraformaldehyde and adsorption to molecular sieves prior to the mechanochemical reaction and using the adsorbed formaldehyde as starting material. This relates to mineral adsorption of formaldehyde on the early Earth, which has been discussed as a prebiotic “sink” for formaldehyde [43]. These reactions were performed in a planetary ball mill in 20 mL stainless steel grinding bowls equipped with an air-tight lid under an oxygen-free atmosphere.

**Figure 6:**
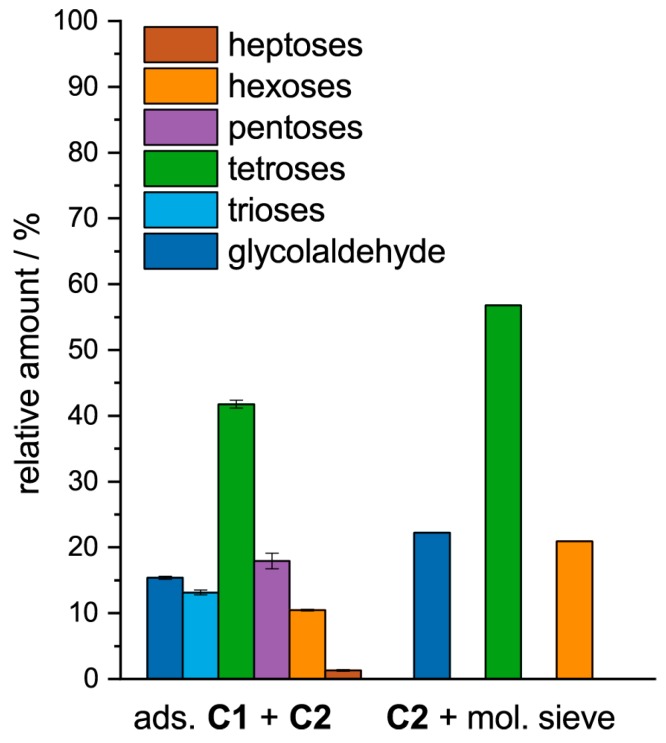
Product distribution of the mechanochemical aldol reaction in a planetary ball mill at 400 rpm after 90 min starting from glycolaldehyde, 20 mol% calcium hydroxide and (a) molecular sieves with adsorbed formaldehyde or (b) dry molecular sieves.

When the reaction was carried out solely with adsorbed formaldehyde and 20 mol% calcium hydroxide, no conversion was observed. However, when glycolaldehyde was added (1:1), monosaccharides were formed (Figure 6). In comparison, we added dry molecular sieves to the reaction of glycolaldehyde and calcium hydroxide where we found only the even-numbered carbon chain length showing that the changing product distribution is not due to the molecular sieves. In the reaction with both glycol- and formaldehyde additionally trioses, pentoses and heptoses were formed in significantly higher amounts, as has been observed previously in the reaction with only glycolaldehyde due to retroaldol reactions (Supplementary Materials). Therefore, we conclude that formaldehyde as the C1 building block is incorporated in this reaction. When the reaction was carried out not in equimolar amounts of formaldehyde and glycolaldehyde, but with catalytic amounts of glycolaldehyde (5 mol%), monosaccharide formation occurred. However, in the same reaction time only traces were detected.

This indicates that the first step of the formose reaction, the dimerization of formaldehyde via an umpolung reaction [54,55], does not proceed under these conditions. Formaldehyde is incorporated as part of the aldol reaction network not only when equimolar amounts of glycolaldehyde are used, but also if minor amounts are already present. These can be accounted for by several suggested sources for the prebiotic occurrence of glycolaldehyde [42,43,47]. It has to be noted, that the initial concentrations of glycolaldehyde and glyceraldehyde on the early Earth were lower than the concentrations experimentally used in this study, which influences the rate of product formation, but not the intrinsic reaction rate constants.

## 4. Conclusions

In summary, we showed that the formation of carbohydrates is not exclusively feasible under an aqueous environment but also establishes under dry conditions, even with a higher selectivity towards unbranched monosaccharides. When glycolaldehyde and glyceraldehyde or formaldehyde are in contact with a basic catalyst, such as minerals or the surrogate calcium hydroxide higher sugars of complex composition form, among them those of biological relevance, such as ribose. Further, we have presented proof that by using a mechanic energy source, the nonaqueous synthesis of sugars is kinetically accelerated and that its product distribution is altered in favor of aldoses and ketoses. Based on those results, we deem it is likely that scenarios of dry and wet cycles, meteoritic impacts and lithospheric activity contributed to the origin of a complex carbohydrate reaction network with the aid of mineral catalysts. The given example of a solid phase reaction, therefore, widens the narrow scope of identified reactions feasible under conditions devoid of water in the context of the origin(s) of life.

## Supplementary Materials

Additional information is available online at https://www.mdpi.com/2075-1729/9/2/52/s1.
