# Supplementary material for: Prebiotic Sugar Formation Under Nonaqueous Conditions and Mechanochemical Acceleration
Source: Life (Basel). 2019 Jun 20;9(2):52. doi: 10.3390/life9020052 (PMC6617062; doi:10.3390/life9020052)
Supplement: Supplementary file 1 [file life-09-00052-s001.pdf]

life

MDPI

Supporting Information

Prebiotic Sugar Formation Under Nonaqueous Conditions and Mechanochemical Acceleration

Saskia Lamour, Sebastian Pallmann, Maren Haas and Oliver Trapp*

Table of Contents

1. Experimental Procedures 2
1.1 Instrumentations: 2
1.1.1 Vortex mixer 2
1.1.2. Ball milling 2
1.1.3. Infrared temperature sensor 2
1.1.4. Gas chromatography 2
2. Data Evaluation 3
2.1 Identification of Carbohydrates 3
2.2 Effective carbon number (ECN) 3
2.3 Determination of Relative Reaction Rates 4
3. Temperature Dependence for Sugar Formation under Nonaqueous Conditions 4
4. Repeatability Study 4
5. Catalyst Loading 5
6. Occurrence and isomerization of trioses 6
7. Tabular Data for Depicted Figures 7
8. References 9

1. Experimental Procedures

1.1 Instrumentations:

1.1.1 Vortex mixer

The vortex mixer Lab Dancer by VWR (VWR International GmbH, Darmstadt, Germany) was used for mixing solids.

1.1.2. Ball milling

For mechanochemically promoted reactions, the oscillatory ball mill *CryoMill* by Retsch (Retsch GmbH, Haan, Germany) was employed equipped with an adapter to fit up to four 5 mL stainless steel ball mill jars each filled with one 7 mm stainless steel ball. In addition, the planetary ball mill *Pulverisette 7* by Fritsch (Fritsch GmbH, Idar-Oberstein, Germany) was used with two 20 mL stainless steel grinding bowls equipped with gassing lids and filled with ten 10 mm stainless steel balls.

1.1.3. Infrared temperature sensor

Temperatures of reaction mixtures within the ball milling jar were determined immediately after stopping the grinding and usage of the infrared temperature sensor VOLTCRAFT IR 260-8S by Conrad (Hirschau, Germany).

1.1.4. Gas chromatography

GC analysis of carbohydrate mixtures was performed on a TraceGC Ultra system coupled to either a PolarisQ MS (quadrupole-ion mass spectrometer [MS]) (Thermo Scientific, San Jose, California, USA) operated by Xcalibur software 1. SR1. Injections used a split/splitless injector in split mode at 250 °C. Flame ionization detection was co-recorded with MS data and operated under carbon-correction at 250 °C.

2. Data Evaluation

2.1 Identification of Carbohydrates

Individual carbohydrates and bulks were detected and relatively quantified using the protocol by Trapp and co-workers.[1] Identification is based on retention times and MS spectra of standard mixtures. Exemplary chromatograms for each of the presented reactions in comparison to the standard mix are given in Figure S1. Due to possible *E*/*Z* isomerism, carbohydrate analytes can show two signals.

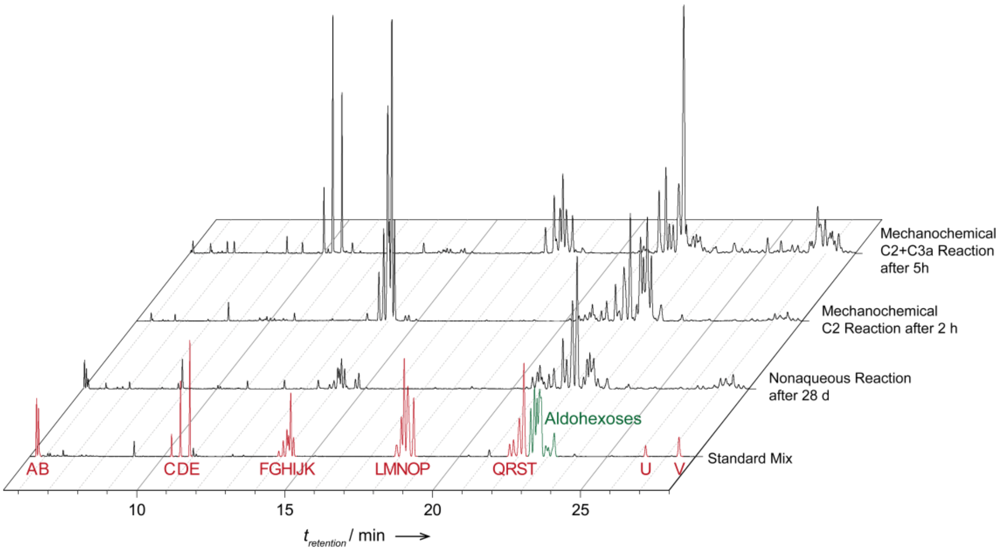

Figure S1. Comparative GC chromatograms for the presented reactions and the standard mix. For method details see experimental section. The given letters denote the following carbohydrates: A/B) glycolaldehyde (**C2**), C/D) glyceraldehyde (**C3a**), E) dihydroxyacetone (**C3b**), F/H) erythrose, G/J) threose, I/K) erythrulose, L/M) xylose, L/N) lyxose, N) arabinose, O) xylulose, O) ribulose, P) ribose, Q/S) tagatose, R/S) psicose, T) sorbose, T) fructose, U/V) galactoheptose. Ketohexoses (Q,R,S,T) and aldohexoses can be identified by their characteristic fragments: 319 m/z and 379 m/z for aldoses and ketoses, respectively.

Distinct **C3** and **C4** constitutional carbohydrate isomers can be identified unambiguously. Observed **C3** carbohydrates are glyceraldehyde (**C3a**), dihydroxyacetone (**C3b**); **C4** carbohydrates are erythrose, threose, erythrulose. Due to overlapping peaks, not all isomers of **C5**, **C6** and **C7** can be unequivocally distinguished. From comparison with the unbranched standards, it is likely, though, that the mixtures contain all known unbranched carbohydrates.

2.2 Effective carbon number (ECN)

To account for different response factors, the effective carbon numbers of monosaccharides and the internal standard were calculated from literature values.[2]

Table 1. Calculated ECN values of monosaccharides.

Compound	ECN
glycolaldehyde	5.29
trioses	8.98
tetroses	12.67
pentoses	16.36
hexoses	20.05
phenyl-β-D-glucopyranoside	20.76

2.3 Determination of Relative Reaction Rates

Using OriginPro 2018G the kinetic data was fitted using linear or cubic polynomial functions. The reaction rates were determined as the first derivative of the molar amount over time for selected time periods.

3. Temperature Dependence for Sugar Formation under Nonaqueous Conditions

The reaction rate of the sugar formation under nonaqueous conditions is temperature dependent. We investigated the conversion for different temperatures and times. The results are given in Figure S2. Higher temperatures facilitate the successive aldol reactions for the formation of higher carbohydrates in a shorter time. Therefore, it is necessary to freeze samples in liquid nitrogen until they can be derivatized and analyzed by GC-MS. For aldol or formose reactions performed in water, the authors, therefore, want to highlight that bias is introduced to samples when employing lyophilization during work-up procedures. Under such conditions, further reaction is possible due to presence of a basic catalyst.

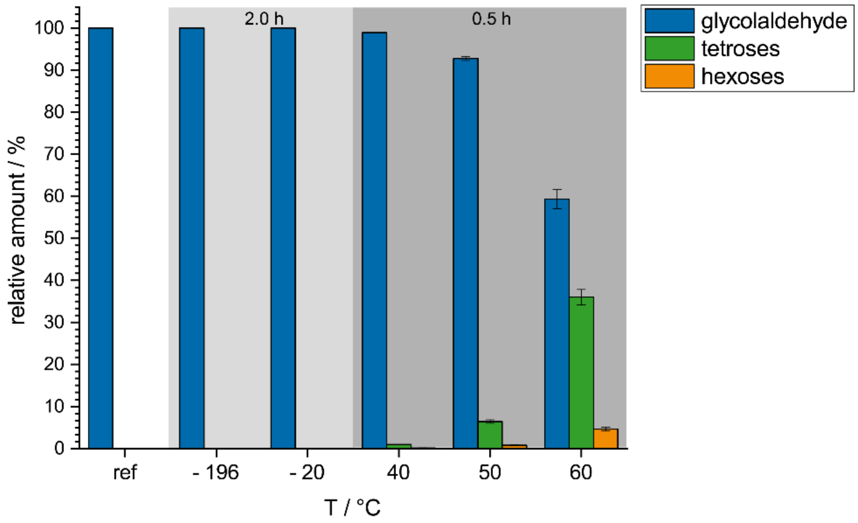

Figure S2. Product distribution of carbohydrate formation under nonaqueous conditions for different temperatures and times. Whereas already after 0.5 h reaction progress can be determined for elevated temperatures, low temperatures do not show any conversion even after 2.0 h. Samples, thus, can be stored for some time under low temperatures. In contrast to the general derivatization protocol described in the experimental procedure section, hydroxylamine hydrochloride was used instead of *O*-ethylhydroxylamine hydrochloride for the experiments of two-hour reaction time.

4. Repeatability Study

All experiments were performed in parallel repetitions. The following data depicts the results of two sets (1 and 2) of one reaction performed one week apart and analyzed with two separately prepared derivatization solutions (A and B). Each GC measurement was run twice. The deviations of observed areas correspond to the error bars. For reaction conditions see reaction setup #2 of the experimental section. Reaction times were 60 min. Based on the repeatability study, we assess the percentage error of the relative frequency of carbohydrates to be at maximum 5 %.

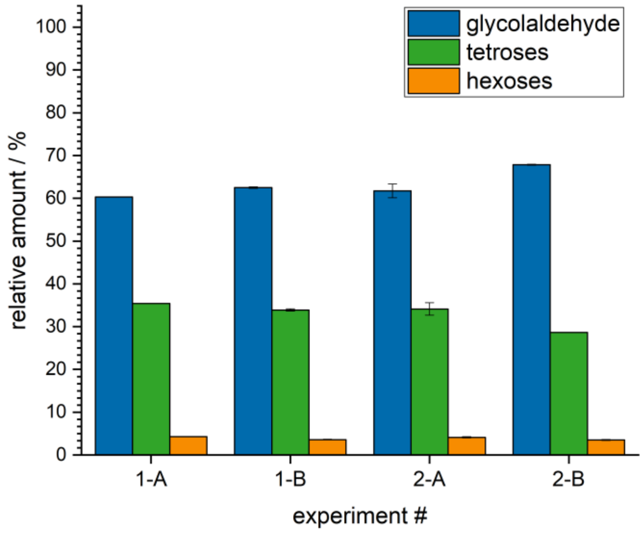

Figure S3. Repeatability study for one reaction performed twice each time one week apart (denoted as 1 and 2) and analyzed with two separately prepared derivatization solutions (A and B). Each GC analysis was run twice. Deviations of areas correspond to error bars. Reaction conditions of the experiment studied are described in the experimental section under reaction setup #2. Reaction times were 60 min.

5. Catalyst Loading

We investigated the catalyst loading in terms of product distribution and substrate consumption (**C2** – glycolaldehyde) under conditions given in the experimental section for reaction setup #2. Reaction times were 90 min. Our investigations show that the reaction rate is dependent on the catalyst loading. We, therefore, argue that even small amounts of $Ca(OH)_2$ are sufficient to trigger the aldose reactions allowing access to the formose reaction network of more complex sugars. It can be envisioned that the slower generation of higher carbohydrates gives way to specific serial reactions with other reactants like amines. In our studies presented here we characteristically employ catalyst loading typical of the formose reaction studied in literature to allow for comparability. Our data, further, implies that with 20 mol% catalyst loading the **C2** consumption reaches saturation.

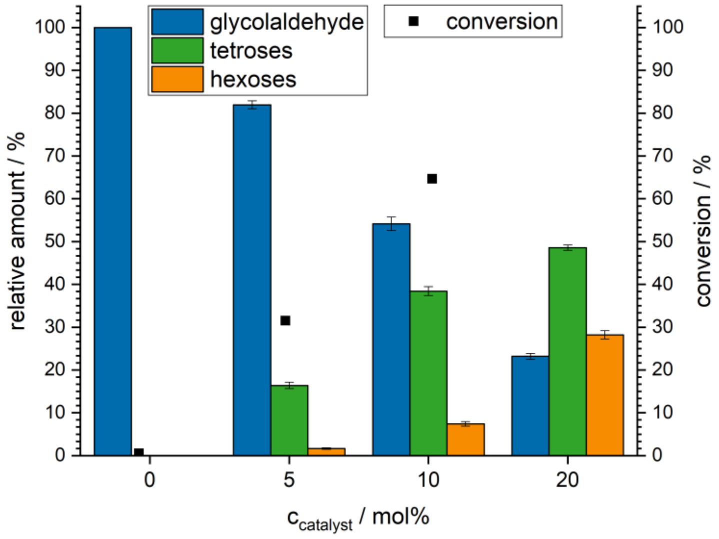

Figure S4. Dependency of product distribution and substrate consumption (**C2** – glycolaldehyde) on catalyst loading. Catalyst loading is in reference to glycolaldehyde monomer.

6. Occurrence and isomerization of trioses

When reactions with the C2 building block were conducted, due to retroaldol reactions of the formed monosaccharides, also trioses were detected in trace amounts. In **figure S5** is the extracted ion chromatogram ($M^{+\bullet}$: *m/z* 277) in the typical area of trioses depicted. Over time glyceraldehyde was formed and after 120 min the isomerization to dihydroxyacetone was observed. Further reactions of these built up heptoses.

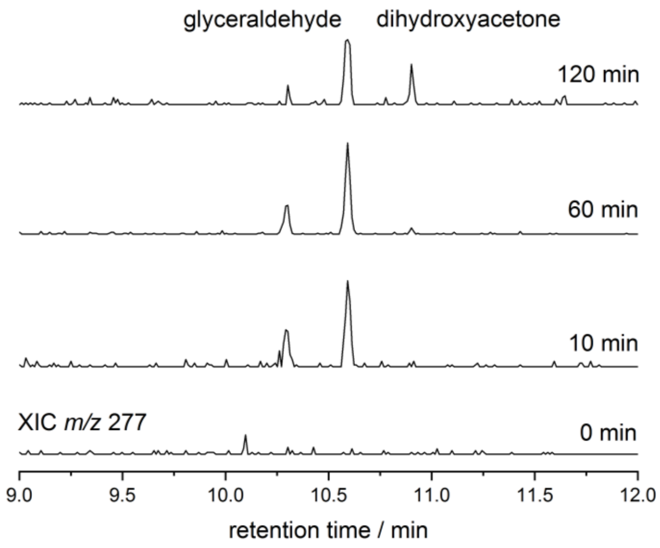

Figure S5: Extracted ion chromatogram (*m/z* 277) of the triose area after different reaction times of C2+$Ca(OH)_2$ reaction in the oscillatory ball mill.

In **Figure S6** differentiated examination of trioses and the comparable interaction of glycolaldehyde, glyceraldehyde and dihydroxyacetone are depicted.

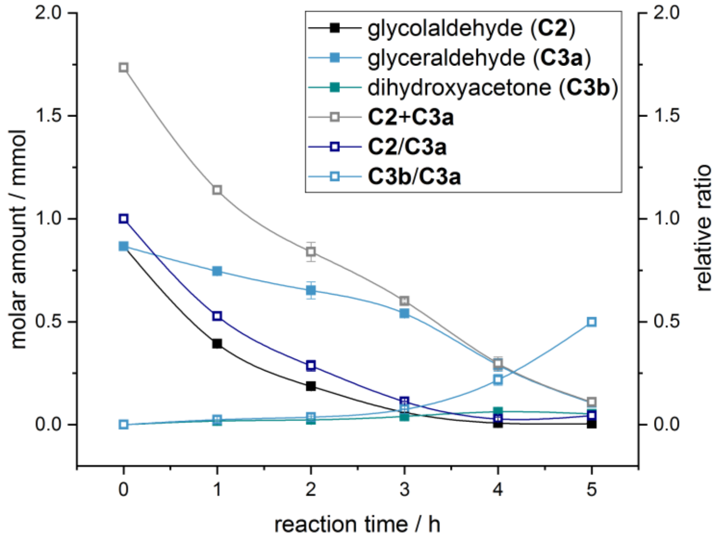

Figure S6: Molar amount of glycolaldehyde and trioses as well as the relative amount of glycolaldehyde or dihydroxyacetone with glyceraldehyde up to 5 h of C2+C3a+$Ca(OH)_2$ reaction in the oscillatory ball mill.

7. Tabular Data for Depicted Figures

Table 2. Relative amounts of sugar mixtures starting from glycolaldehyde with different mineral catalysts or pure calcium hydroxide as base used in mechanochemical sugar formation (**Figure 1**).[1] Error analysis is based on double determinations.

mineral	glycolaldehyde [%]	tetroses [%]	hexoses [%]
brucite	92.7 ± 1.0	6.8 ± 0.9	0.5 ± 0.1
montmorillonite	85.3 ± 2.6	14.2 ± 2.7	0.6 ± 0.2
portlandite	44.9 ± 1.3	42.9 ± 1.0	12.2 ± 0.4
pure calcium hydroxide	35.8 ± 4.7	50.5 ± 3.0	13.7 ± 1.8

[1] Oscillatory ball mill, 30 Hz, 90 min, 20 mol% catalyst.

Table 3. Molar amounts of sugar mixtures starting from glycolaldehyde with 20 mol% calcium hydroxide in mechanochemical sugar formation (**Figure 2**).[1] Error analysis is based on double determinations.

time [min]	glycolaldehyde [mmol]	tetroses [mmol]	hexoses [mmol]	heptoses [mmol]
0	2.08 ± 0.00	0.00 ± 0.00	0.00 ± 0.00	0.00 ± 0.00
2.5	1.88 ± 0.00	0.03 ± 0.00	0.00 ± 0.00	0.00 ± 0.00
5	1.66 ± 0.00	0.14 ± 0.00	0.00 ± 0.00	0.00 ± 0.00
7.5	1.36 ± 0.01	0.26 ± 0.00	0.02 ± 0.00	0.00 ± 0.00
10	0.99 ± 0.20	0.45 ± 0.06	0.04 ± 0.01	0.00 ± 0.00
20	0.76 ± 0,10	0.54 ± 0.05	0.05 ± 0.01	0.00 ± 0.00
30	0.59 ± 0.04	0.57 ± 0.02	0.07 ± 0.00	0.00 ± 0.00
60	0.41 ± 0.04	0.62 ± 0.00	0.13 ± 0.01	0.00 ± 0.00
90	0.31 ± 0.03	0.59 ± 0.00	0.18 ± 0.01	0.01 ± 0.00
120	0.08 ± 0.01	0.42 ± 0.01	0.27 ± 0.01	0.01 ± 0.00

[1] Oscillatory ball mill, 30 Hz.

Table 4. Molar amounts of sugar mixtures starting from glycolaldehyde and glyceraldehyde with 20 mol% calcium hydroxide in mechanochemical sugar formation (**Figure 3**).[1] Error analysis is based on double determinations.

time [h]	glycolaldehyde [mmol]	trioses [mmol]	tetroses [mmol]	pentoses [mmol]	hexoses [mmol]	heptoses [mmol]
0	0.87 ± 0.00	0.87 ± 0.00	0.00 ± 0.00	0.00 ± 0.00	0.00 ± 0.00	0.00 ± 0.00
1	0.39 ± 0.01	0.76 ± 0.00	0.16 ± 0.00	0.06 ± 0.00	0.02 ± 0.00	0.01 ± 0.00
2	0.19 ± 0.01	0.68 ± 0.04	0.19 ± 0.01	0.09 ± 0.02	0.04 ± 0.01	0.02 ± 0.00
3	0.06 ± 0.00	0.58 ± 0.01	0.13 ± 0.01	0.12 ± 0.00	0.11 ± 0.00	0.05 ± 0.00
4	0.01 ± 0.00	0.35 ± 0.03	0.03 ± 0.00	0.13 ± 0.00	0.22 ± 0.01	0.08 ± 0.00
5	0.00 ± 0.00	0.16 ± 0.00	0.01 ± 0.00	0.14 ± 0.00	0.29 ± 0.00	0.01 ± 0.00

[1] Oscillatory ball mill, 30 Hz.

Table 5. Molar amounts of ribose and relative ratio of ribose relating to all pentoses during the reaction starting from glycolaldehyde and glyceraldehyde with 20 mol% calcium hydroxide in mechanochemical sugar formation (**Figure 4**).[1] Error analysis is based on double determinations.

time [h]	ribose [µmol]	ribose/pentoses [%]
0	0.0 ± 0.0	--
1	4.8 ± 0.2	8.2 ± 0.0
2	6.9 ± 0.5	7.4 ± 0.0
3	10.9 ± 0.2	8.9 ± 0.0
4	14.9 ± 0.3	11.1 ± 0.0
5	16.1 ± 0.1	11.8 ± 0.0

[1] Oscillatory ball mill, 30 Hz.

Table 7. Relative amounts for the mechanochemical reaction[1] and the reaction in water (140 mM, 40 °C) of glycolaldehyde with 20 mol% $Ca(OH)_2$ after 30 min (**Figure 5**).

medium	glycolaldehyde [%]	tetroses [%]	hexoses [%]	heptoses [%]
mechanochemical	53	42	5	0
in water	1	12	81	6

[1] Oscillatory ball mill, 30 Hz.

Table 8. Relative amounts for the mechanochemical aldol reaction in a planetary ball mill at 400 rpm after 90 min (**Figure 6**).

molecular sieve	glycol-aldehyde [%]	trioses [%]	tetroses [%]	pentoses [%]	hexoses [%]	heptoses [%]
molecular sieves with adsorbed formaldehyde	15	13	42	18	10	1
dry molecular sieves	22	0	57	0	21	0

Table 9. Product distribution of carbohydrate formation for different storage temperatures and times starting from glycolaldehyde with 20 mol% calcium hydroxide in a glass vial (**Figure S2**). Error analysis is based on double determinations.

time [h]	temperature [°C]	glycolaldehyde [%]	tetroses [%]	hexoses [%]
0	23	100.0 ± 0.0	0.0 ± 0.0	0.0 ± 0.0
2	-196	100.0 ± 0.0	0.0 ± 0.0	0.0 ± 0.0
2	-20	100.0 ± 0.0	0.0 ± 0.0	0.0 ± 0.0
0.5	40	98.9 ± 0.1	0.9 ± 0.1	0.1 ± 0.0
0.5	50	92.8 ± 0.5	6.4 ± 0.4	0.8 ± 0.1
0.5	60	59.3 ± 2.3	36.0 ± 1.8	4.6 ± 0.5

Table 10. Repeatability study for the mechanochemical reaction starting from glycolaldehyde with 20 mol% calcium hydroxide (**Figure S3**).[1] Error analysis is based on double determinations.

experiment #	glycolaldehyde [%]	tetroses [%]	hexoses [%]
1-A	60.3 ± 0.1	35.4 ± 0.1	4.3 ± 0.1
1-B	62.5 ± 0.2	33.9 ± 0.3	3.6 ± 0.1
2-A	61.8 ± 1.6	34.1 ± 1.4	4.1 ± 0.1
2-B	67.9 ± 0.1	28.6 ± 0.1	3.5 ± 0.1

[1] Oscillatory ball mill, 30 Hz, 60 min.

Table 11. Repeatability study for the mechanochemical reaction starting from glycolaldehyde with 20 mol% calcium hydroxide (**Figure S4**).[1] Error analysis is based on double determinations.

catalyst [mol%]	glycolaldehyde [%]	tetroses [%]	hexoses [%]
0	100.0 ± 0.0	0.0 ± 0.0	0.0 ± 0.0
5	82.0 ± 0.9	16.4 ± 0.7	1.6 ± 0.2
10	54.2 ± 1.6	38.4 ± 1.0	7.4 ± 0.5
20	23.2 ± 0.7	48.6 ± 0.6	28.2 ± 1.0

[1] Oscillatory ball mill, 30 Hz, 90 min.

8. References

1. Haas, M.; Lamour, S.; Trapp, O. Development of an advanced derivatization protocol for the unambiguous identification of monosaccharides in complex mixtures by gas and liquid chromatography. *J. Chromatogr. A* **2018**, *1568*, 160-167, doi:https://doi.org/10.1016/j.chroma.2018.07.015.
2. Scanlon, J.T.; Willis, D.E. Calculation of Flame Ionization Detector Relative Response Factors Using the Effective Carbon Number Concept. *J. Chromatogr. Sci.* **1985**, *23*, 333-340, doi:10.1093/chromsci/23.8.333.
